# Increased prevalence of fungemia in Medina, Saudi Arabia

**DOI:** 10.3389/fepid.2023.1180331

**Published:** 2023-05-09

**Authors:** Aiah M. Khateb, Fadwa S. Alofi, Abdullah Z. Almutairi

**Affiliations:** ^1^ Department of Medical Laboratory Technology, Collage of Applied Medical Science, Taibah University, Medina, Saudi Arabia; ^2^ Infectious Diseases Department, King Fahad Hospital, Medina, Saudi Arabia; ^3^ Microbiology Laboratory, King Fahad Hospital, Medina, Saudi Arabia

**Keywords:** Candida, fungemia, non-Candida albicans, resistance, women

## Abstract

**Background:**

The prevalence of fungal infection is increasing globally due to an increase in the immunocompromised and aging population. We investigated epidemiological changes in fungemia in one of the major centers in Medina over seven years period with 87,447 admissions.

**Methods:**

Retrospective search of records for causative agents of fungemia in inpatients at King Fahad Hospital (KFH) in 2013–2019. Fungal-positive blood cultures, demographic, and treatment data were extracted.

**Results:**

A total of 331 fungemia episodes proven by blood culture were identified in 46 patients. The annual prevalence of fungemia increased from 0.072 in 2013 to 1.546 patients per 1,000 in 2019. The mean age of fungemia episodes was 56 years, and 62% of episodes occurred in females. Samples from central blood incubated aerobically yielded the highest fungemia rate, accounting for 55% (*n* = 182). Among yeast species, *Candida parapsilosis* was responsible for the highest number of episodes 37% (*n* = 122), followed by *Candida glabrata* (32%; *n* = 107), *Candid albicans* (29%; *n* = 94), and *Cryptococcus neoformans* (1%; *n* = 4). Among molds, *Lichtheimia* (*Absidia)* species was the most common (1%; *n* = 3). Yeast-like fungi *Trichosporion mucoides* accounted for (0.003% *n* = 1). The use of antifungal treatment has increased (96%) over the years (2013–2019). An increase in resistance rate of 2% was found in *C. albicans* and C. *glabrata*. The most prevalent comorbidity was renal disease (24.2%).

**Conclusions:**

*C. parapsilosis* was the leading cause of fungemia. The association of renal disease with increased candidemia was alarming. This study is a fundamental resource to establish management policies for fungal infection in the region.

## 1. Introduction

There are over 300 million individuals affected by fungal infections worldwide. Recent multi-national analysis has estimated that fungal infections are killing 1.5 million cases annually (1). Invasive fungal infections (IFI) are rising due to many factors, including pathogenic strains, underlying medical conditions, early or late diagnosis, the choice of therapy, and drug resistance. With a delay in diagnosis and treatment or not achieving effective therapeutic drug levels, IFI may develop into a chronic fungal infection (CFI). CFI tends to increase mortality if left untreated and increase resistance if not treated correctly. The most common causative agents of IFI are *Candida albicans, Aspergillus fumigatus, Cryptococcus neoformans, Pneumocystis jirovecii,* endemic dimorphic fungi, and *Mucormycetes* (2, 3). IFI is proven when tissue damage due to fungal components is detected by histopathologic assessment and/or when the etiologic agent is isolated by culture from clinical, sterile samples, such as blood, tissue, or cerebrospinal fluid (4, 5). IFI varies according to the geographical and populational background. Certain epidemiological reports and a few laboratories have adopted updated diagnostic methods, especially for mold detection in Saudi Arabia. However, the number of populations at risk for IFI increases in the country, including those with chronic kidney disease, lower respiratory infections, and diabetes (6). Based on the life expectancy at birth in Saudi Arabia (1990–2010), the longevity of Saudis will increase by an average of 5.3 years in both male and female subjects (7, 8). In this study, we investigated the characteristics of bloodstream fungal infections (fungemia) in the largest hospital in Medina, King Fahad Hospital (KFH).

## 2. Materials and methods

### 2.1. Study design and ethical approval

Approval of the study from the local IRB/ethics committee (IRB 590) was obtained from the institutional review board and general directorate of health affairs in Medina, Ministry of Health, Saudi Arabia (#H-03-M-084). The need for informed consent was waived by the committee due to the study application form and its observational-retrospective nature.

### 2.2. Inclusion and exclusion criteria

**Figure 1:**
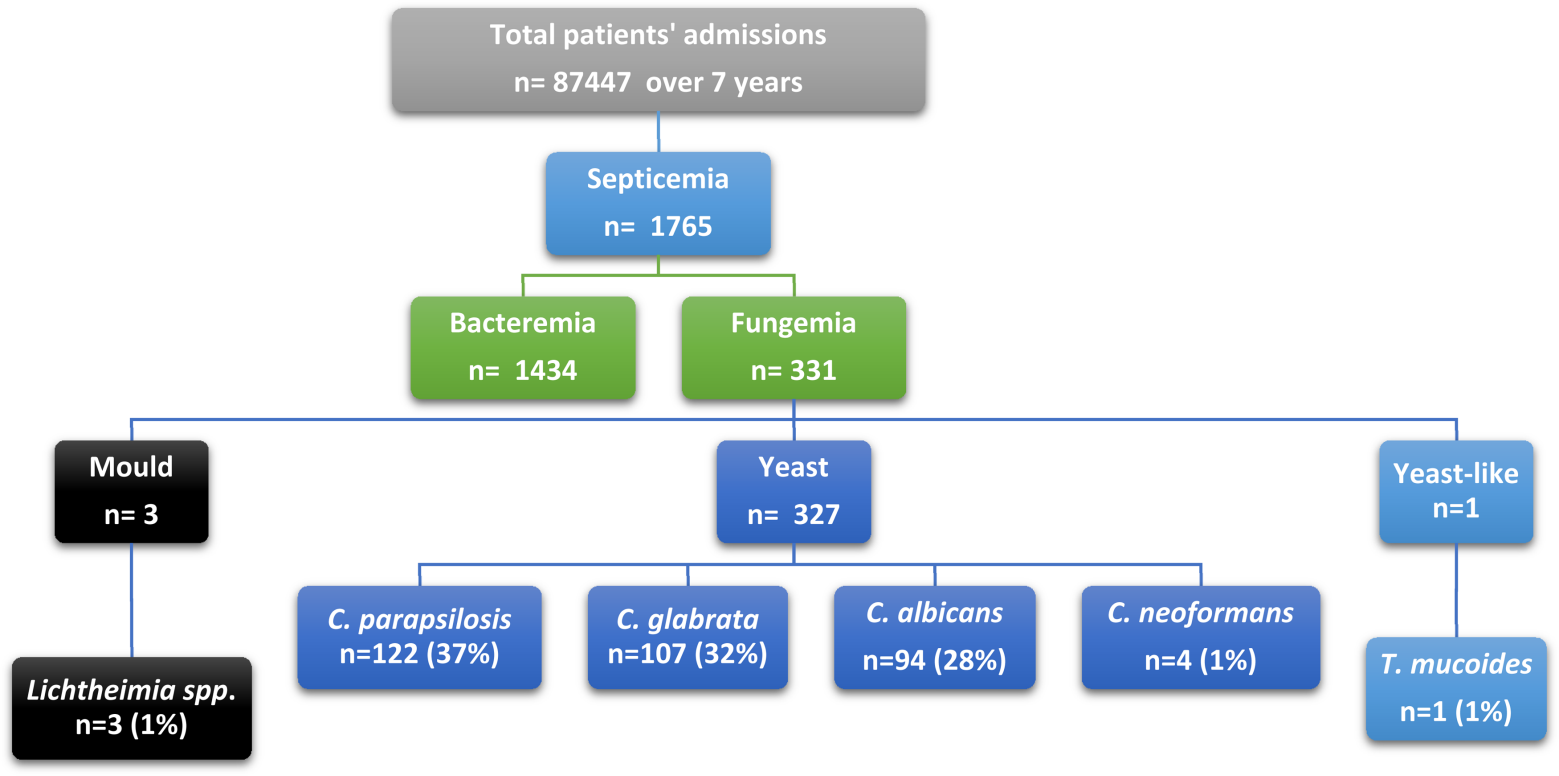
Flow diagram of the study design and selection criteria.

All hospitalized patients from all departments with positive fungal blood cultures were included from July 2013 to December 2019 (Figure 1). Data were reviewed from the hospital database for patients with positive blood culture. These cases would be classified as proven according to the European Organization for Research and Treatment of Cancer/Mycosis Study Group (EORTC/MSG) if T2 *Candida* panel was used and if complex image data and clinical analysis was performed (5). The targeted population data included all episodes of patients with fungemia, their initial diagnosis, age, comorbid conditions, identified species, antifungal therapy, and resistance profile. An episode of fungemia was defined as the isolation of any pathogenic species of fungi from at least one blood culture specimen from a patient with signs and symptoms of infection. If a patient had multiple admissions, they were included in the study as new episodes (Figure 1).

### 2.3. Microbiological laboratory methods

Blood samples from peripheral and central lines were collected when infection signs and symptoms were noted. The samples were cultured aerobically and anaerobically using the fully automated BacT/Alert and BACTEC-fx Microbial Detection System until a signal-positive alarm was sounded or for a maximum of 5 days (bioMérieux, Durham, NC, USA). BACTEC™ Myco/F lytic culture vials for fungi was used for high risk cases. Positive blood culture bottles were processed for further identification using Gram staining. If fungal elements were seen in Gram stain, samples were sub-cultured on Sabroud Dextrose agar plates (SDA) and incubated at 25–30°C for 24 h for 3 weeks in a mycology incubator (Forma Scientific Incubator, Germany). Further conventional identification and sensitivity testing were performed for yeasts using the VITEK 2 system (bioMérieux, Marcy-L'Étoile, France) according to the manufacturer's instructions. Antifungal susceptibility reporting criteria were interpreted based on the updated guidelines of the CLSI (resistance, sensitivity, and intermediate resistance) (9). Identification of molds was made using macroscopic and microscopic examination with lactophenol cotton blue stain, but no mold antifungal susceptibility was performed.

### 2.4. Statistical analysis

Numerical data were repted as mean and mode ± standard deviation, and categorical data frequencies and percentages were reported using Microsoft 365 Excel. Depending on the data distributions, risk groups and comparative risk factors of candidemia were tested using multivariate regression. A *P* value ≤ 0.05 was considered statistically significant. The annual prevalence of fungal infections was calculated as patients with fungemia were counted as one case per year in the data analysis. Patients' episodes were counted every time treatment was changed or new positive culture was identified. The prevalence rate of fungemia based on hospital admissions was calculated by dividing the number of patients with a fungal infection by the number of total admissions: $\mathrm{prevalence}\;\mathrm{rat}e=(n/\mathrm{totalpopulation})\times 10^{n}$. The change ratio of fungemia episodes over the years was calculated by dividing the number of episodes from the year before the desired year to be calculated. The overall increased ratio was calculated by dividing the number of episodes in 2019 by the number of episodes in 2013.

## 3. Results

### 3.1. Demographic characteristics of fungemia patients

Over a 7-year period, 46 patients had fungemia at KFH (26 females, 20 males). These patients had a total of 331 episodes. The mean age of patients with fungemia episodes in the study was 56 years, and 62% of fungemia episodes were found in females. More details on the population's demographics, including mean, standard deviation, standard error, median, mode, standard deviation, confidence level (95%), upper confidence level, lower confidence level 95%, and age groups, are shown in Table 1. By using regression analysis, there was overall statistical significance regarding fungemia predominance in females compared to males (*P*-value = 0.01). The most common fungal causative agents among 331 fungemia episodes were *Candida parapsilosis* (*n* = 122; 36.85%), *Candida glabrata* (*n* = 107; 32.32%), *Candida albicans* (*n* = 94; 28.39%), *Cryptococcus neoformans* (*n* = 4; 1.20%), *Lichtheimia* sp. (*n* = 3; 0.90%), and *Trichosporon mucoides* (*n* = 1; 0.30%), as shown in Table 1. Among young adults (18–30 years), we found positive blood culture for *Candida parapsilosis*, *Cryptococcus neoformans*, *Lichtheimia* sp., and *Trichosporon mucoides* species, but no *Candida glabrata*, as shown in Supplementary Figure S2*.* In adults (31–50 years) and seniors (61–100), we found *Candida parapsilosis, Candida glabrata*, and *Candida albicans* to be the causative agents in fungemia, but no molds were seen. The most common associated conditions and risk factors for acquiring fungemia were renal diseases (24.2%), gastrointestinal tract diseases (GIT) (16%), cardiovascular system diseases (CVS) (14%), respiratory diseases (12.6%), oncology (8%), accidents (5%), burns (4.2%), fever (3.6%), sepsis (3.3%), unknown factors (3.3%), dermatomyositis (2.1%), hypersensitivity angiitis (1.8%), and Turner's syndrome (0.9%) Supplementary Figure S2.

**Table 1:** The population's fungal causative agent's species and demographics including: mean, standard deviation, standard error, median, mode, standard deviation, confidence level (95%), upper confidence level, lower confidence level 95% and age groups in years.

Demographics	Number of episodes (*n*)	Percentage %
**Gender**		
Male	127	38%
Female	207	63%
**Age (years) mean (56)**		
Mean	56.4	
Standard error	1.1	
Median	55	
Mode	41	
Standard deviation	20.2	
Confidence level (95.0%)	2.2	
Upper confidence level	58.6	
Lower confidence level	0	
**Age groups in years**		
Young adults (18–30 years)	30	9%
Adults (31–50 years)	146	45%
Seniors (61–100	155	47%

### 3.2. Prevalence of fungemia

Fungemia prevalence has dramatically increased over the study period. Fungemia prevalence was 0.072 patients per 1,000 in 2013 and 1.546 patients per 1,000 in 2019, as demonstrated in Table 2. The fungemia episode change ratio over the years showed a gradual increase. The highest increase of 2.9 was detected in 2017–2018. Overall, the number of fungemia episodes increased 54.6-fold in 2013–2019, as shown in Table 2.

**Table 2:** Fungemia prevalence rate in KFH admission, incidence rate in septicemia patients, change ratio of fungemia episodes over the years.

Counts/Years	Number of patients	Number of episodes	Hospital admissions	Prevalence based on number of patients/1,000	Prevalence Rate of the fungemia	Increase over year	Increase ratio of fungemia
2013	1	3	13,816	0.072	0.001	**2013–2014**	1.7
2014	4	9	12,787	0.312	3.128	**2014–2015**	2.75
2015	3	16	12,475	0.240	0.24	**2015–2016**	0.54
2016	5	44	12,818	0.390	39.008	**2017–2018**	2.96
2017	5	24	12,646	0.395	39.538	**2018–2019**	2.31
2018	10	71	11,267	0.887	88,75,477	**2013–2019**	54.6667
2019	18	164	11,638	1.546	1.55 × 10^15^	–	–
Totals	46	331	87,447	–	–	–	–

### 3.3. Fungemia patterns in age and sex

Changes in the age patterns over seven years were noted as follows. In 2013, there were only female episodes in patients under 40 years old. Between 2014 and 2015, the incidence of fungemia episodes in females increased 2.5-fold, and only 4 male episodes were recorded in total. In 2016, the number of episodes also increased 2.9-fold compared to 2015, and no male episodes were recorded. In 2017, the number of episodes dropped 2.5-fold in females, while male cases recorded 7 episodes. Interestingly, starting from 2018, the number of episodes was almost identical in both males and females (36 and 35 episodes, respectively) (Supplementary Figure S1). Moreover, a new age group of young adults (under the age of 30) appeared in females only. The young adult group persisted till 2019 in females, and only one case was recorded in a young male adult. Compared to 2018, the number of fungemia episodes was 2.5-fold higher in females in 2019. Also, in males, fungemia episodes recorded a 2.1-fold increase, which was the highest number over a 7-year period of the study.

Fungal infection ratio was high in females (1.7) (females: *n* = 207; males: *n* = 124). Across age groups, the highest number of episodes was among adult females (40–65 years; 103 females), while the number of male episodes peaked in the senior group (>65 years; 66 males). In adults, the highest episode number was caused by *Candida parapsilosis* (*n* = 77), followed by *Candida glabrata* (*n* = 49) and *Candida albicans* (*n* = 24). However, in seniors, *Candida glabrata* was responsible for the highest number of episodes (*n* = 58), followed by *Candida albicans* (*n* = 51), and *Candida parapsilosis* (*n* = 13) was responsible for the lowest number of episodes (Supplementary Figure S2). In the emerging incidence of fungemia episodes in young adults, most infections were caused by *Candida parapsilosis* (*n* = 32), followed by *Candida albicans* (*n* = 19), and few episodes were caused by *Cryptococcus neoformans* (*n* = 4)*, Lichtheimia* species (*n* = 3), and *Trichosporon mucoides* (*n* = 1)*.* No *Candida glabrata* episodes were seen in young adults. Nonetheless, there was no statistical significance comparing the ages of patients individually or by the three age groups and causative organisms (*P*-value 0.50 and 0.12, respectively).

### 3.4. Causative agents of fungemia episodes

C*andida* species was the principal causative agent responsible for fungemia (97.5%). At KFH, most fungemia episodes were caused by yeast species, of which *Candida parapsilosis* was responsible for the highest number of episodes (37%; *n* = 122), followed by *Candida glabrata* (32%; *n* = 107), *Candid albicans* (29%; *n* = 94), and *Cryptococcus neoformans* (1%; *n* = 4). *Lichtheimia* species was the only mold detected (1%; *n* = 3). Yeast-like fungi were represented by *Trichosporion mucoides*, accounting for 0.003% of cases (*n* = 1). All patients had a single causative agent detected by culture in every episode, except for two patients in 2019 (male and female) in whom *C. albicans* was the causative agent, followed by *C. parapsilosis*. Non*-albicans* strains were the leading cause of candidemia across all years, accounting for 71% of all episodes. Moreover, *Candida albicans* was the causative agent responsible for 29% of all candidemia episodes (Supplementary Figures S3, S4).

The number of species causing fungemia varied according to culture method and sample source. Over the study period, the highest number of fungemia isolates was found in central blood incubated aerobically, accounting for 55% (*n* = 182) of all episodes (Supplementary Table S1). The main causative agent in this sample type was *Candida albicans,* followed by *Candida parapsilosis, Candida glabrata,* and *Cryptococcus neoformans.* The second most common type of samples with fungemia was found in peripheral blood incubated aerobically (Supplementary Table S1). The main causative agent in this sample category was *Candida parapsilosis,* followed by *Candida albicans* and *Candida glabrata.* Samples from central blood incubated anaerobically were obtained from 3 cases caused by yeast-like fungi *(Trichosporon mucoides)* and one case caused by molds (*Lichtheimia species)*. Finally, peripheral blood samples incubated anaerobically yielded the lowest fungal growth and only grew *Candida glabrata* species (Supplementary Table S1).

### 3.5. Candidemia episodes

There was a noticeable shift in candidemia species, as the number of non-*albicans Candida* species (NAC) was higher (71%; *n* = 229) than that of *C. albicans* (29%; *n* = 94) (Supplementary Figure S4). This shift was statistically significant (*P-*value = 0.032). During 2013–2016, there was only one episode caused by *C. albicans.* However, the number of NAC almost doubled over the same period (2013 = 0, 2014 = 7, 2015 = 16, 2016 = 44). In 2017, *C. albicans* emerged again, causing almost half of NAC cases (6 of 17 episodes). *C. albicans* episodes continued to increase gradually, and in the next year, it caused more episodes than NAC (2018 = 37 and 31 episodes, respectively). However, in 2019, the number of NAC almost doubled compared to cases caused by *C. albicans* (50 and 114 episodes, respectively). The distribution of *C. albicans* and NAC from 2013 to 2019 is shown in Supplementary Figure S4. Candidemia was found in 63% (*n* = 206) of females and 37% (*n* = 122) of males (Table 3). The highest number of episodes was found in adults (31–50 years; 63%; *n* = 207), followed by seniors (51–100 years; 27%; *n* = 90) and young adults (15–30 years; 9%; *n* = 31). Risk factors and associated conditions were analyzed using multivariant regression of the probability of the increasing number of episodes caused by *C. albicans vs.* those caused by NAC as a causative agent of candidemia at KFH. The top five common risk factors were found in patients with renal diseases (26.2%; *n* = 86), GIT diseases (20.4%; *n* = 67), respiratory diseases (14.02%; *n* = 46), CVS diseases (12.5%; *n* = 41), and oncology (7%; *n* = 24). Only three factors were statistically significant: gender, fever, and sepsis (*P*-values were 0.053, 0.017, and 0.017, respectively; Table 3).

**Table 3:** Precentage of risk factors and multivariant regression of probability of increasing episodes of *Candida albicans* verses *non-Candida albicas* as causetive agent of candidemia at KFH.

Regression Statistics summery for risk factors			* *	* *
Multiple R	0.433			
R Square	0.188			
Adjusted R Square	0.148			
Standard error	0.419			
Observations	323			
Regression F	4.749			
Significance F	2.92E-08			
Intercept *P*-value	0.063			
Variable	Number of episodes	Percentage	Standard error	*P*-value
**Age**				
Young adults 18–30:	31	9%	0.001	0.351
Adults 31–50:	207	63%		
Senior 51–100:	90	27%		
**Gender**				
Female	206	63%	0.007	0.053^*^
Male	122	37%		
Unknown	8	2.4%	0.296	0.112
GIT	67	20.4%	0.253	0.118
Renal	86	26.2%	0.249	0.267
CVS	41	12.5%	0.251	0.093
Respiratory	46	14.02%	0.250	0.134
Oncology	24	7%	0.134	0.250
Burns	0	0%	0	0
Fever	12	3.6%	0.259	0.017^*^
Accident	18	5.4%	0.267	0.839
Hypersensitivity angiitis	5	1.5%	0.311	0.206
Dermatomyositis	7	2.1%	0.295	0.344
Sepsis	12	3.6%	0.259	0.017^*^
Turner's syndrome, unspecified	3	0.9%	0.349	0.363

^*^ Statistically significant.

### 3.6. Fungemia treatment and antifungal resistance

**Figure 2:**
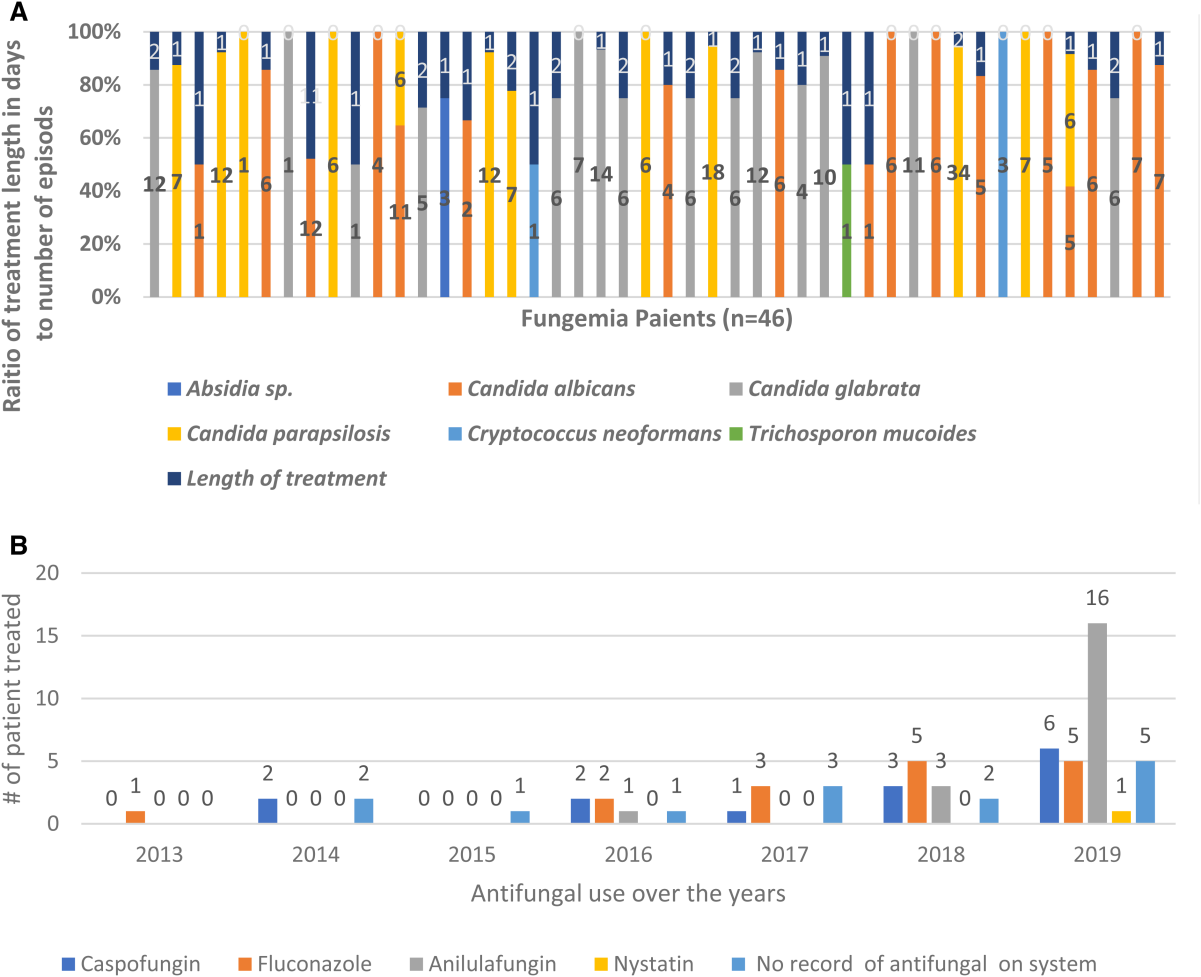
Increase use of antifungal treatment over years 2013–2019. Overall use of antifungal treatment has increased over 7 years in response to the increase of fungemia patients. (**A**) Raito of treatment duration to the number of episodes for each fungemia patient. (**B**) Antifungal use over 7 years showing the number of patients treated each year.

Our data showed that 70% (32 out of 46 patients) of all hospital fungemia patients received antifungal treatment in 2013–2019. Unfortunately, hospital recorders did not report any antifungal treatment for 30% (*n* = 14) of fungemia patients. The most selected antifungal medication was anidulafungin (39%; *n* = 20), followed by fluconazole (31%; *n* = 16), caspofungin (28%; *n* = 14), and nystatin (2%; *n* = 1). The use of antifungals has increased dramatically (96%) due to the increase in the number of fungemia episodes over 7 years (Figure 2). In 2013, only one patient was treated with one antifungal drug.

Antifungal sensitivity assays tested for 5-fluorocytosine, amphotericin B, caspofungin, fluconazole, micafungin, and voriconazole resistance in all but 26 episodes (8%). Most isolates were susceptible (96%; *n* = 293), 1.9% (*n* = 6) of isolates were intermediate-resistant, and 1.6% (*n* = 5) of isolates were resistant. The highest resistance was seen in *C. glabrata* species. Four isolates were intermediate-resistant to caspofungin and fluconazole (2 isolates each). Additionally, 2 *C. glabrata* isolates were highly resistant to fluconazole. The resistance rate was 2% in *C. albicans*. One isolate was intermediate-resistant to amphotericin B, and 2 isolates were resistant to fluconazole and voriconazole. The resistance rate to *C. glabrata* was 2.3%. In *C. parapsilosis* and *C. neoformans*, there were no resistant isolates detected (Supplementary Table S3).

## 4. Discussion

Fungemia is a serious threat characterized by high mortality. The systemic infection may present as an extreme flu-like syndrome with severe symptoms, such as acute confusion, chronic fatigue, unusual discharge, and impaired wound healing. This retrospective study showed a dramatic increase in fungemia cases in Medina, with a prevalence of 1.546 patients per 1,000 individuals. It is important to reduce the prevalence of fungemia using protective measures for hand hygiene, catheter management, and antibiotic management. Al-Hedaithy et al. have reported that 50.3% of episodes were caused by *Candida albicans* and *C. dubliniensis*, followed by *C. tropicalis, C. parapsilosis*, *C. glabrata*, *C. krusei,* and *C. famata*, in Riyadh University hospital (10). More recent studies have also found that *C. albicans* only represented 33% of candidemia. However, there was a noticeable decrease in *C. albicans* cases and an increase in the number of NAC cases (11, 12). In Riyadh hospitals, the NAC increase was led by *C. tropicalis*, followed by *C. glabrata* (11, 13–17). However, in Medina, *C. parapsilosis* was the leading cause of NAC increase. No *C. aurus, C. dubliniensis, C. krusei,* or *C. famata*. This change in the distribution of disease reflected the growing prevalence of NAC in Saudi Arabia. On an international level, the SENTRY investigators, which used data from 135 medical centers in 39 countries, collected data from a twenty-year surveillance for *Candida* species infections in general in 1997–2016 (18). They found that *C. albicans* was the predominant species in Europe (52.5%) and the least common in North America. *C. glabrata* was most common in North America (24.3%) (18). *C. parapsilosis* was the predominant species in Latin America. *C. tropicalis* was the predominant species in the Asia Pacific region (14.1%) (18). In an earlier study by the same group, *C. albicans* was the most frequent species responsible for bloodstream fungal infections (1996–1999) in all geographic regions except for the US, where *C. glabrata* was more common (19). These variations represent differences in populations, treatment regimens, and other encountered risk factors (20, 21).

We identified only one mold isolate of *Lichtheimia* (*Absidia*) species, which was seen in a young adult patient. *Lichtheimia* spp*.* belongs to mucormycota, which are saprotrophic opportunistic pathogens. *Lichtheimia* spp*.* causes aggressive pulmonary, CNS, rhinocerebral, or cutaneous infections with impaired immunity and rarely infects the blood. Early diagnosis is crucial due to frequent dissemination (22). Yeast-like fungi *Trichosporon* spp*.* is an opportunistic emerging fungal pathogen in immunosuppressed patients (23). Although it is found in soil, water, and normal flora of the human skin, upper respiratory tract, and GIT, several reports have described *Trichosporon* spp*.* causing fungemia (24).

Sex may play a role as a risk factor, since females, as compared to males, were more affected by fungemia annually across the study period. However, more focused studies are needed to confirm this. Baharoon et al. have identified that the use of broad-spectrum antibiotics was a major risk. This information was not accessible in the electronic records of this study population (13). We found that fungemia occurred more frequently in patients with renal, GIT, CVS, and respiratory diseases, oncology, accidents, burns, fever, and sepsis. The study data were based on the primary diagnosis by the physicians upon patients' admission; therefore, the prognosis could not be confirmed and address morbidity and mortality. Other studies have linked the increase in NAC to the increasing use of antifungals, particularly fluconazole (21). Fluconazole was prescribed in most patients (85%; *n* = 40). Almost all isolates were susceptible (96%; *n* = 293), 1.9% (*n* = 6) of isolates were intermediate-resistant, and 1.6% (*n* = 5) of isolates were resistant to fluconazole and voriconazole. This highlights the need to review the selection of empiric antifungal therapy (21).

The epidemiology of fungal diseases is constantly evolving. Many factors play a role in increasing fungal infection susceptibility, including the geographical location, the population at risk, the virulence of fungal species, increase in longevity, medical and treatment advancement, invasive procedures, large-scale health events, and seasonal epidemics. The currently available diagnostic tests in the majority of hospitals are insufficient to give a complete picture of the actual status. Thus, the current epidemiologic trends should support investigational efforts, diagnosis, and treatment. To our knowledge, this study is the first to describe fungemia cases in Medina. The direct medical cost of fungal diseases globally was estimated by the Centers for Disease Control and Prevention (CDC) at $7.2 billion. Adding indirect social costs, the costs would be higher as many fungal infections are underdiagnosed or unreported (6). Investing in training and raising awareness of common causative fungal agents will guide clinicians and technologies in improving diagnosis. It is essential to develop a clinical prediction tool for preventive screening for fungal infections to educate and guide the diagnostic process and use of antifungals.

## Data Availability

The raw data supporting the conclusions of this article will be made available by the authors, without undue reservation.
