# Supplementary material for: Increased prevalence of fungemia in Medina, Saudi Arabia
Source: Front Epidemiol. 2023 May 9;3:1180331. doi: 10.3389/fepid.2023.1180331 (PMC10910952; doi:10.3389/fepid.2023.1180331)
Supplement: Supplementary file 1 [file Datasheet1.docx]

**Supplementary figures and tables**

**Figure S1. Number of fungemia episodes of by gender (male; female) in different age groups in years.** Using regression analysis there was overall statistical significance of fungemia seen in female episodes compared to male. (*P*-value 0.01).

**Figure S2.** **Fungal causative agents’ distribution among age groups.** There was no statistical significance comparing ages of patients individually or by groups *P*-value 0.50 and 0.12 respectively. Mold isolates were only seen in blood cultures of young adults but not in adults and seniors. No C*andida galbrata* isolates were found in young adults.

**Figure S3. Fungemia risk factors and underlying conditions.** Over the 7-year period the most common five underlining conditions were renal (24.2%), gastrointestinal tract diseases (GIT) (16%), Cardiovascular system (CVS) (14%), respiratory (12.6%), oncology (8%), accidents (5%), burns (4.2%), fever (3.6 %), sepsis (3.3%), unknown (3.3%), dermatomyositis (2.1%), Hypersensitivity angiitis (1.8%), and Turner's syndrome (0.9%).

**Figure S4. The distribution of *Candida albicans* and *non-albicans* species from 2013 to 2019.** Only one episode of *C. albicans* was seen 2013-2019. The number of episodes have gradually increased to *non-albicans* species significantly 2017- 2019. *P*-value=0.032. Multiple R=0.795, R Square= 0.633, Adjusted R Square=0.559, Standard Error= 13.924, Observations =7, Significance F=0.032, F= 8.635, Standard Error= 0.146.

**Table S1 Fungal organism isolated from different sample source.**

| **Sample source and incubation** | ***Absidia sp.*** | ***Candida albicans*** | ***Candida glabrata*** | ***Candida parapsilosis*** | ***Cryptococcus neoformans*** | ***Trichosporon mucoides*** | **Grand Total** | **Percent %** |
| --- | --- | --- | --- | --- | --- | --- | --- | --- |
| **Blood (aerobic) central** |  | 65 | 49 | 64 | 4 |  | 182 | 55 |
| **Blood (aerobic) peripheral** |  | 22 | 17 | 39 |  |  | 78 | 23.6 |
| **Blood (anaerobic) central** | 3 | 6 | 24 | 19 |  | 1 | 53 | 16 |
| **Blood (anaerobic) peripheral** |  |  | 17 |  |  |  | 17 | 5.14 |
| **CVP TIP** |  | 1 |  |  |  |  | 1 | 0.3 |

**Table S2 Antifungal susceptibility results for *Candida* and *Cryptococcus* species and resistance rate.** Antifungals tested included 5- Fluorocytosine, Caspofungin, Amphotercin B, Fluconazole, Micafungin, and Voriconazole.

| **Antifungal susceptibility testing** | **5- Fluorocytosine** | **Amphotercin B** | **Caspofungin** | **Fluconazole** | **Micafungin** | **Voriconazole** | **Grand Total** | **Resistance rate %** |
| --- | --- | --- | --- | --- | --- | --- | --- | --- |
| ***C. albicans* tested isolates** | 11 | 16 | 16 | 16 | 13 | 15 | 87 | 2.29 |
| **Intermediate** |  | 1 |  |  |  |  | 1 |  |
| **Resistance** |  |  |  | 1 |  | 1 | 2 |  |
| **Sensitive** | 11 | 15 | 16 | 15 | 13 | 14 | 84 |  |
| ***C. glabrata* tested isolates** | 14 | 18 | 18 | 15 | 17 | 17 | 99 | 2.02 |
| **Intermediate** |  |  | 2 | 2 |  |  | 4 |  |
| **Resistance** |  |  |  | 2 |  |  | 2 |  |
| **Sensitive** | 14 | 18 | 16 | 11 | 17 | 17 | 93 |  |
